# Superficial Femoral Artery Endothelial Responses to a Short-Term Altered Shear Rate Intervention in Healthy Men

**DOI:** 10.1371/journal.pone.0113407

**Published:** 2014-11-21

**Authors:** Julia O. Totosy de Zepetnek, Tena L. Jermey, Maureen J. MacDonald

**Affiliations:** Department of Kinesiology, McMaster University, Hamilton, Ontario, Canada; Academia Sinica, Taiwan

## Abstract

In animal and in-vitro models, increased oscillatory shear stress characterized by increased retrograde shear-rate (SR) is associated with acutely decreased endothelial cell function. While previous research suggests a possible detrimental role of elevated retrograde SR on endothelial-function in the brachial artery in humans, little research has been conducted examining arteries in the leg. Examinations of altered shear pattern in the superficial femoral artery (SFA) are important, as this vessel is both prone to atherosclerosis and leg exercise is a common form of activity in humans. Seven healthy men participated; bilateral endothelial-function was assessed via flow-mediated-dilation (FMD) before and after 30-minute unilateral inflations of a thigh blood pressure cuff to either 75 mmHg or 100 mmHg on two separate visits. Inflation of the cuff induced increases in maximum anterograde (p<0.05), maximum retrograde (p<0.01), and oscillatory shear index (OSI) (p<0.001) in the cuffed leg at both inflation pressures. At 100 mmHg the increases in SR were larger in the retrograde than the anterograde direction evidenced by a decrease in mean SR (p<0.01). There was an acute decrease in relative FMD in the cuffed leg alone following inflation to both pressures. These results indicate that in the SFA, altered SR profiles incorporating increased retrograde and OSI influence the attenuation in FMD after a 30-minute unilateral thigh-cuff inflation intervention. Novel information highlighting the importance of OSI calculations and assessments of flow profiles add to current body of knowledge regarding the influence of changes in SR patterns on FMD. Findings from the current study may provide additional insight when designing strategies to combat impaired vascular function in the lower extremity where blood vessels are more prone to atherosclerosis in comparison to the upper extremity.

## Introduction

Endothelial dysfunction is widely accepted as a precursor to the development of atherosclerosis, may contribute to later stage cardiovascular disease (CVD) [1], and is predictive of cardiovascular events in healthy individuals as well as in those with existing CVD [2]. Wall shear stress is an important determinant of endothelial cell function [3]. *In vitro* studies indicate that high shear stress induces atheroprotective endothelial gene expression (e.g. increased production of endothelial nitric oxide synthase), while low shear stress stimulates an atherogenic phenotype (e.g. increased production of endothelin 1, thrombomodulin, vascular cell adhesion molecule-1 [VCAM-1], intercellular adhesion molecule-1 [ICAM-1]) [4], [5]. The pattern of shear stress also contributes to the regulation of vascular structure and function. In cultured cells and animal models, laminar unidirectional shear stress maintains normal endothelial structure and function, while stasis, turbulent flow, or oscillatory shear stress induces an atherogenic state and is associated with inflammation, atherosclerotic lesions, lower numbers of stress fibers, and high endothelial permeability [6].

Shear stress can be approximated non-invasively *in vivo* by shear rate (SR  =  blood velocity/lumen diameter). One assumption for SR calculations is that blood velocity moves in a parabolic manner. It has been demonstrated in animals that anterograde blood velocity moves in a parabolic-like shape [7], [8], however one study conducted in dogs reported a non-parabolic profile of retrograde blood velocity [7]. A recent study in humans examined the shape of anterograde and retrograde blood velocity profiles through the femoral artery using the relationship between mean (MBV) and peak (PBV) blood velocity across the vessel lumen; they defined a parabolic velocity when mean velocity is half the peak velocity (MBV/PBV  = 0.5) [9]. They found mean, anterograde, and retrograde profiles were parabolic in shape at rest (i.e. MBV/PBV ratio not different from 0.5). These findings suggest calculations of mean, anterograde, and retrograde SR are acceptable approximations when evaluating endothelial function via flow-mediated dilation (FMD), and when examining resting SR patterns.

The influence of SR patterns on FMD has not been extensively investigated in humans *in vivo*, and most investigations have focused on the brachial artery. Three human studies in this area suggest increasing anterograde SR acutely enhances brachial artery FMD and can counteract the potential negative influence of elevated retrograde SR, while increased retrograde SR alone acutely impairs brachial artery FMD [10]–[12]. In contrast to the upper extremity, the vasculature of the lower extremity is highly susceptible to atherosclerotic lesion formation [13], [14]. It has been reported that resting SR in the superficial femoral artery (SFA) are lower than those in the brachial artery [15], and that the normal pattern of SR in the SFA includes a larger retrograde component in comparison to the brachial artery [16]. This chronically lower wall shear stress, along with higher turbulence through the SFA due to anatomical structure, has been suggested to contribute to higher incidence of atherosclerosis in comparison to the upper extremity blood vessels [15], [17]. Further, vascular disease risk is often detectable in the blood vessels of the legs rather than the arms (i.e. intermittent claudication, peripheral arterial disease) [18]. One recent study assessing SFA FMD response to induced retrograde SR reported decreased FMD following a 60 mmHg thigh cuff inflation intervention; however the researchers were unable to create an exclusively retrograde shear stimulus as had been done previously in the brachial artery with similar interventions [19]. The purpose of the present study was to extend this work by examining the acute effects of higher cuff inflation pressures (75 and 100 mmHg) designed to induce greater increases in retrograde SR and thereby maximize the impact on SFA endothelial function as measured by FMD. We hypothesized that cuff pressures of 75 and 100 mmHg would induce elevations in retrograde SR that would retain a parabolic flow profile and would impair SFA FMD in young healthy males in a dose dependent manner.

## Methods

### Participants

Nine healthy recreationally active men (age: 26.6±5.9 years; waist circumference: 82.8±5.8 cm; body mass index: 23.8±2.2 kg/m^2^) were recruited from McMaster University in Hamilton, ON, Canada. None of the participants had been diagnosed with CVD or any risk factors leading to the development of CVD such as hypercholesterolemia, hyperlipidemia, or hypertension, and none were on any medications that are known to affect the cardiovascular system. The study procedures were approved by the Hamilton Health Sciences Research Ethics committee in Hamilton, Ontario, Canada, adhered to the Declaration of Helsinki, and all of the participants gave previous written informed consent.

### Experimental Design

Each participant came to the laboratory on two occasions for approximately two hours in a fasted state (≧8 hours), abstained from caffeine and alcohol for ≧12 hours, and abstained from physical activity for a minimum of 24 hours. Both visits were done at the same time of day to eliminate diurnal variation. Assessment was conducted in a quiet, temperature controlled room (22–24°C). SFA FMD was assessed in both legs (cuffed [altered flow intervention] and non-cuffed [control]) before and immediately after a 30-minute unilateral thigh cuff-inflation intervention designed to alter SR.

### Experimental Procedures

#### Flow Mediated Dilation and Shear Rate

FMD is an established and reliable method of assessing endothelial-dependent vascular function and is considered to be primarily nitric oxide mediated in the more commonly assessed brachial artery [20], [21]. There is recent evidence that SFA FMD is also primarily nitric oxide mediated [2], [22]. Prior to the baseline FMD assessments, participants rested in the supine position for at least 15-minutes to ensure stability in resting supine leg blood flow, blood pressure (BP), and heart rate (HR) measures. HR was monitored continuously throughout all testing procedures using a single lead electrocardiogram (ECG; Model ML123, ADInstruments Inc.; Colorado Springs, USA), and supine BP was monitored in triplicate at baseline and immediately following cuff release using an automated blood pressure device (Dinamap, GE Healthcare; Horten, Norway).

At baseline a three-heart cycle brightness mode image and 30-seconds of MBV using pulsed-wave Doppler were recorded of the SFA approximately 3–5 cm distal to the common femoral artery bifurcation using high-resolution ultrasound (System FiVe, GE Medical Systems; Horten, Norway). A large cuff (CC17 Hokanson; Washington, USA) was placed around the upper thigh approximately 10 cm distal to the greater trochanter and instantaneously inflated to 200 mmHg for 5-minutes (E20 Rapid Cuff Inflator, AG 101 Cuff Inflator Air Source, Hokanson; Washington, USA). Upon cuff deflation reactive hyperemic MBV through the SFA was assessed for the first 30-seconds and subsequently three-heart cycle SFA diameter brightness-mode images were digitally stored at standardized time points for 5-minutes following cuff release (45s, 60s, 75s, 90s, 120s, 180s, 240s, 300s) [2].

Images were ECG-gated, collected at a frame rate of 15 frames/s, and were stored for off-line analyses [23]. End diastolic frames were selected from each three-heart cycle image to create a 3-frame stacked digital imaging and communications in medicine file (Sante DICOM Editor, Version 3.1.20, Santesoft; Greece). Stacked SFA end diastolic diameters were analyzed from the near wall to the far wall to include the intima, media, and lumen using custom-designed semi-automated edge-detection software (Artery Measurement System Image and Data Analysis, Tomas Gustavsson; Sweden). Relative FMD was calculated as shown in equation 1:

1. Relative FMD (%)  =  ((Peak – Baseline Diameter)/(Baseline Diameter))*100.
  Sample volume (gate width) encompassed the entire lumen (from intima-to-intima) for MBV measurements using pulsed-wave Doppler. Raw blood velocity profiles were outsourced to a spectral analyzer (Neurovision 500 M TCD, Multigon Instruments; Yonkers, USA). Intensity weighted MBV was determined using fast Fourier transformation and acquired with an analog to digital data acquisition system for offline beat-to-beat analyses (PowerLab 16/35 with LabChart 7 Pro, ADInstruments Inc.; Colorado Springs, USA). Velocity measures were collected at an insonation angle of 68 degrees for all participants. MBV SR was calculated by dividing the blood velocity values by the end diastolic arterial diameters as shown in equation 2 below:
2. SR (1/sec)  =  (8 * Mean Blood Velocity)/(End Diastolic Lumen Diameter)
  Reactive hyperemic SR from 0–30s (SR_AUC_0–30s) after cuff release has been shown to significantly correlate with relative FMD in young adults similar to the correlation between FMD and the commonly assessed area under the SR curve until the time to peak diameter [24]. We therefore used the SR_AUC_0–30s to represent the SR stimuli after cuff release as the full SR response until peak dilation was not technically possible to obtain with our equipment.
  Oscillatory shear index (OSI) was used as an indicator of the magnitude of shear oscillation or shear reversal. For purely oscillatory flow, the OSI attains a maximum value of 0.5. Consistently high values of OSI have been associated with endothelial dysfunction [25]. OSI was calculated as shown in equation 3 below:
3. OSI  =  (|retrograde SR|)/(|retrograde SR| + |anterograde SR|)

#### Altered Shear Intervention

**Figure 1:**
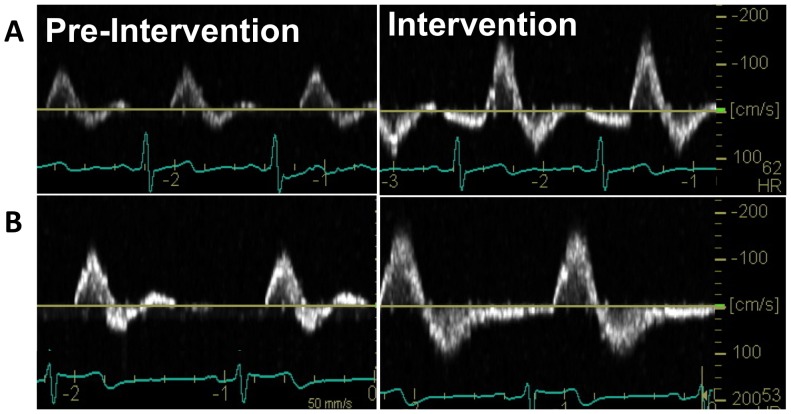
Superficial Femoral Artery Cuff-Inflation Intervention (Doppler Screen Capture). Screen capture of the Doppler velocity profile before and at 30-minute of the (A) 75 mmHg and (B) 100 mmHg cuff inflation in the cuffed leg.

The same large leg cuff used for the assessment of FMD% was used to induce altered SR patterns in the dominant leg. Leg dominance was determined to be the same side as hand dominance. Following baseline diameter and blood velocity measures, the cuff was inflated to 75 mmHg on the first visit and 100 mmHg on the second visit for 30-minutes. The cuff inflation pressures were chosen based on a previous study reporting impaired brachial artery FMD% following 30-minutes of forearm cuff pressure at 75 mmHg [10]. Owing to the larger SFA diameter and in keeping with the aim of creating an exclusive retrograde stimulus, we further selected the 100 mmHg inflation pressure. SFA diameters as well as blood velocity measures were obtained every 5-minutes throughout the 30-minute intervention and stored for off-line analyses (Figure 1). Mean, anterograde, and retrograde SR were determined over a 30-second period every 5-minutes throughout the intervention.

#### Blood Velocity Shape

At baseline and at each data collection time point throughout the 30-minute cuff-inflation intervention, intensity-weighted MBV and PBV were outsourced separately to the same spectral analyzer as described above (Neurovision 500 M TCD, Multigon Instruments; Yonkers, USA; PowerLab 16/35 with LabChart 7 Pro, ADInstruments Inc.; Colorado Springs, USA).

Values for MBV, PBV, and MBV/PBV ratio were determined for mean, anterograde, and retrograde phases of the cardiac cycle during rest. Excess retrograde blood velocity is purposefully created during the cuff inflation intervention, resulting in altered ratios of MBV to PBV that influence the entire cardiac cycle calculations. We therefore calculated MBV, PBV, and MBV/PBV for the anterograde and retrograde components only during the cuff-inflation intervention. The anterograde phase included all anterograde blood velocity and the retrograde phase included all retrograde blood velocity within an entire cardiac cycle. When MBV/PBV  = 0.5 parabolic velocity was present, MBV/PBV  = 1.0 represented a plug-like profile, and MBV/PBV ≈0 represented a sharpened parabolic profile [9].

### Statistics

Statistical analyses were performed using SPSS 17.0 software. Paired t-tests were used to assess the baseline characteristics between the two testing days. Repeated measures analysis of variance was used to determine the effects of time (pre vs. post-intervention) and leg (cuffed vs. non-cuffed) on FMD and SR. Post-hoc t-tests with Bonferroni correction were performed when a significant main or interaction effect was found. Paired t-tests were used to determine the differences in SR and SR patterns at baseline and at 30-minutes of the intervention.

Single sample t-tests were used to determine if MBV/PBV ratios were different from 0.5 at rest and during increased retrograde SR, and paired t-tests were used to determine if MBV/PBV ratios were different between baseline and increased retrograde SR conditions.

## Results

**Figure 2:**
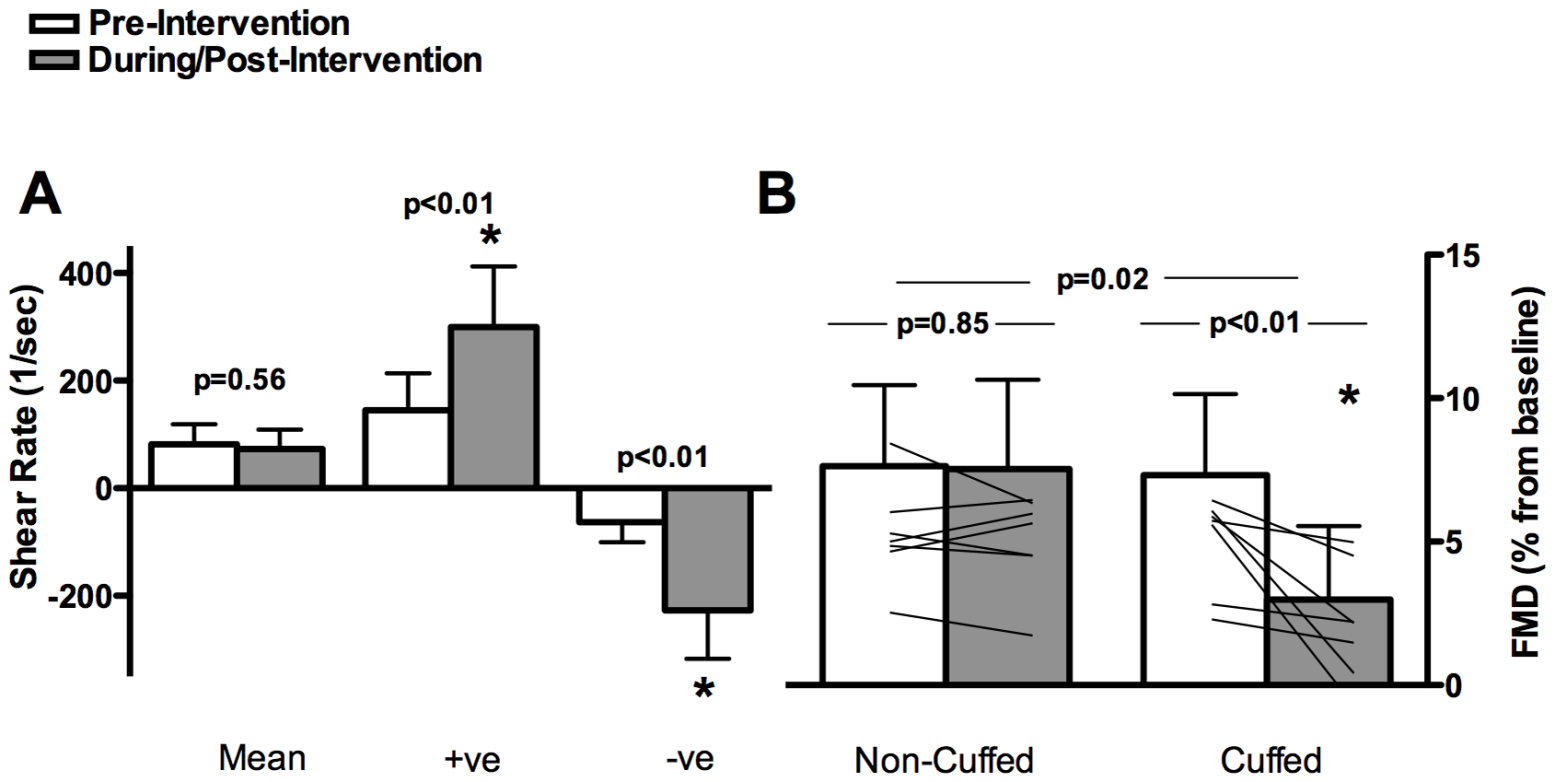
Superficial Femoral Artery 75 mmHg Cuff-Inflation Intervention. On the left (A): mean, anterograde (+ ve), and retrograde (-ve) SR patterns pre- and during the intervention in the cuffed leg. On the right (B): relative FMD before and after the intervention in the cuffed and non-cuffed leg; mean and individual data are presented (n = 7). Error bars represent SD.

**Figure 3:**
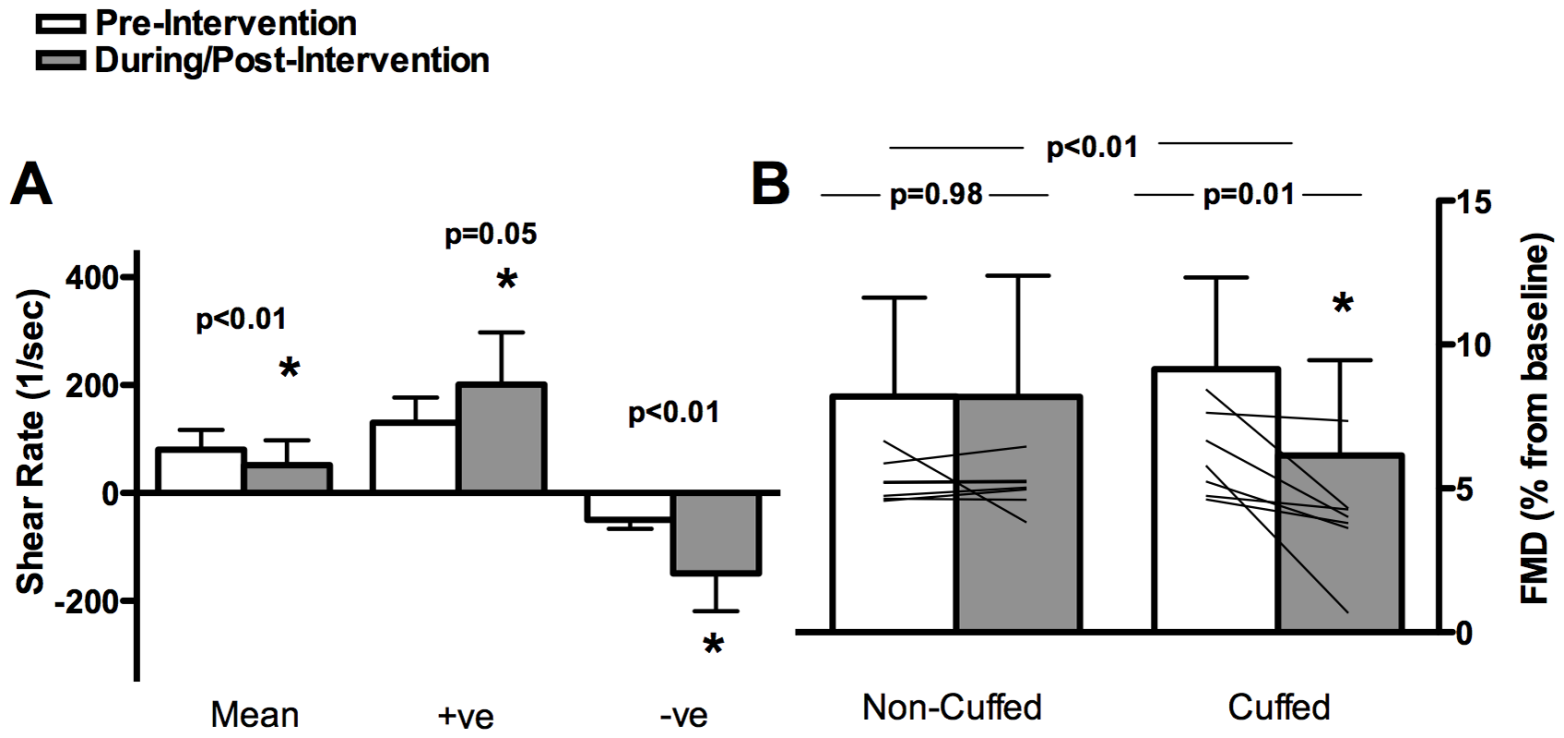
Superficial Femoral Artery 100 mmHg Cuff-Inflation Intervention. On the left (A): mean, anterograde (+ ve), and retrograde (-ve) SR patterns pre- and during the intervention in the cuffed leg. On the right (B): relative FMD before and after the intervention in the cuffed and non-cuffed leg; mean and individual data are presented (n = 7). Error bars represent SD.

Due to discomfort, one participant could not complete the 75 mmHg and another could not complete the 100 mmHg testing session; therefore n = 8 participants completed both testing days, and n = 7 were included in the final analyses. Pre-intervention BP, HR, SR, and FMD were not different across the two testing days (Tables 1 and 2). There were significant increases in peak retrograde (p<0.01, p<0.01) and anterograde (p<0.01, p = 0.05) SR in the cuffed leg but not the control leg during the 75 and 100 mmHg interventions, respectively (Figures 2 and 3). There was no dose response change in SR parameters to the increasing inflation pressure; however during the 100 mmHg cuff pressure intervention the larger increase in retrograde SR vs. anterograde SR resulted in a decrease in mean SR (p<0.01). There was a significant increase in OSI in the cuffed leg only during cuff inflation intervention to both 75 and 100 mmHg (p<0.001; Table 2).

**Table 1:** Supine blood pressure and heart rate.

Parameter	75 mmHg		100 mmHg		
	Before	After	Before	After	p-value
SBP, mmHg	129±21	134±19	127±8	130±10	0.78
DBP, mmHg	75±14	79±16	69±6	73±7	0.31
MAP, mmHg	98±21	102±27	88±6	92±8	0.25
HR, bpm	65±10	67±13	66±11	66±11	0.65

Data are for participants before and after both interventions (n = 7).

Values are mean±SD. Abbreviations: SBP  =  systolic blood pressure; DBP  =  diastolic blood pressure; MAP  =  mean arterial pressure; HR  =  heart rate. P value refers to paired t-tests between the pre-intervention values between the two testing days. No differences were found pre- to post-intervention at either cuff pressures.

**Table 2:** Superficial femoral artery shear rate and flow-mediated dilation characteristics.

Parameter	Cuffed			Non-Cuffed		
	Before	After	p-value	Before	After	p-value
75 mmHg						
Baseline D, mm	6.5±0.4	6.4±0.4	0.506	6.7±0.5	6.7±0.6	0.390
Peak SR, s^−1^	810±218	910±158	0.231	793±354	942±176	0.153
SR_AUC_ 0–30, 10^3^	5.32±1.73	6.08±2.31	0.372	5.80±3.41	6.72±2.06	0.219
OSI	0.30±0.06	0.43±0.03	<0.001	0.24±0.07	0.27±0.07	0.148
SFA FMD, mm	0.47±0.19	0.19±0.17	0.018	0.50±0.19	0.48±0.20	0.636
100 mmHg						
Baseline D, mm	6.0±0.6	6.1±0.7	0.756	6.0±0.6	6.0±0.6	0.794
Peak SR, s^−1^	958±127	866±264	0.320	903±307	1001±372	0.455
SR_AUC_ 0–30, 10^3^	5.57±1.74	5.18±1.20	0.609	5.71±2.30	6.48±3.15	0.437
OSI	0.28±0.05	0.42±0.06	<0.001	0.27±0.07	0.29±0.07	0.450
SFA FMD, mm	0.58±0.23	0.40±0.26	0.012	0.51±0.27	0.51±0.32	0.993

Data is of participants before and after both interventions in cuffed and non-cuffed leg (n = 7).

Values are mean ± SD. D =  diameter; SR_AUC_ 0–30 =  shear rate area under curve for 30sec; OSI  =  oscillatory shear index; SFA FMD  =  superficial femoral artery flow mediated dilation. P value refers to paired t-tests pre-vs. post-cuff inflation intervention. No differences were found between baseline values on the two testing days, or between cuffed vs. non-cuffed leg baseline measures.

A significant interaction (time x leg) indicated a decrease in FMD% in the cuffed leg only after both the 75 and 100 mmHg interventions (p<0.05, Figures 2 and 3). Baseline SFA reproducibility of FMD% assessment between testing days agrees with previous literature; the ICC and CV was 0.57 and 25%, respectively.

At baseline the mean ratio of MBV/PBV was greater than 0.5 indicating a blunted parabolic shape; the retrograde ratio was less than 0.5 indicating a sharpened parabolic shape; the anterograde ratio was not different from 0.5 indicating a parabolic shape. At 30-minutes of the cuff inflation intervention the retrograde ratio was less than 0.5 and the anterograde ratio was greater than 0.5. Both anterograde and retrograde ratios increased from baseline to 30-minutes of the cuff intervention (Table 3).

**Table 3:** Superficial femoral artery hemodynamics.

Parameter	Before	p-value	At 30min	p-value	p-value
		vs. 0.5		vs. 0.5	pre vs. post
Cardiac Cycle					
MBV (cm s^−1^)	6.4±2.6		−		−
PBV (cm s^−1^)	10.1±4.6		−		−
MBV/PBV	0.65±0.14	<0.001	−	−	−
Anterograde					
MBV (cm s^−1^)	11.0±4.5		20.0±9.3		<0.001
PBV (cm s^−1^)	22.2±8.9		35.6±15.1		0.001
MBV/PBV	0.49±0.05	0.600	0.55±.05	0.003	0.006
Retrograde					
MBV (cm s^−1^)	−4.5±2.4		−15.1±7.3		<0.001
PBV (cm s^−1^)	−12.1±5.7		−34.7±14.7		<0.001
MBV/PBV	0.37±0.05*	0.001	0.42±0.07*	<0.001	0.034

Data is responses to 30min cuff-inflation at 75 mmHg and 100 mHg (n = 16).

Values are mean±SD. Abbreviations: MBV  =  average mean blood velocity; PBV  =  average peak blood velocity; “P value vs. 0.5″ refers to single sample t-tests between the MBV/PBV ratio and 0.5 (the blood velocity profile is a perfect parabola). “P value pre vs. post” refers to paired t-tests between pre-intervention (before) and at 30-min of intervention (at 30min). *p<0.001, significantly different from anterograde MBV/PBV ratio.

## Discussion

Our results provide novel information about changes in OSI as well as the shape of the flow profile while supporting the concept that a brief period of enhanced oscillatory SR acutely attenuates FMD% in the legs of humans. Our results also challenge previous conclusions that these FMD% changes can be exclusively attributed to retrograde SR changes induced by cuff inflation interventions. Consistent with previous reports examining both the upper and lower limbs [10], [19], our unilateral leg cuff procedures induced altered SFA SR patterns that resulted in acute decreases in FMD% in the cuffed leg only. Despite using higher cuff pressures than previous work, we were not able to induce an exclusive retrograde SR alteration. In the cuffed leg we observed changes in both anterograde and retrograde SR at both cuff inflation pressures, however we observed larger increases in retrograde SR at the 100 mmHg cuff pressure, evidenced by a decrease in mean SR (Figure 3). No alterations in flow patterns were observed in the non-cuffed leg.

Decreases in mean and increases in oscillatory SR have been shown to be detrimental to endothelial cells [26], [27], and are observed in arteries where the propensity for atherosclerosis is higher [1], [15], [28]. Although OSI cannot differentiate between uniaxial and multidirectional flows, high OSI has been is thought to trigger atherosclerosis [29]. A recent review assessing the relationship between various methodologies of wall shear stressors and atherosclerosis reported that while a few previous studies have shown no relationship between wall shear stress and disease, the majority of evidence points to the involvement of OSI in atherosclerotic progression [30]. Our findings of an increase in OSI following the cuff-inflation interventions and subsequent decrease in FMD% may support the proposed influence of OSI on endothelial cells.

Our intention was to induce a retrograde stimulus without affecting anterograde SR through the SFA at rest, thereby replicating the stimulus applied to the brachial artery in a previous study [10]. As both retrograde and anterograde SFA SR were augmented in response to our 30-minute thigh cuff inflations, we cannot comment on the effects of isolated increases in retrograde SR on endothelial health. Since anterograde SR is considered to be atheroprotective [6], it is likely the acute increases in anterograde SR observed with the present protocol provided some cardio-protective effect to the SFA. It is possible that a more pronounced decrease in SFA endothelial function could occur following an exclusive retrograde stimulus. In contrast to previous work in the upper extremity [10], it seems it is not possible to induce an exclusive retrograde SR stimulus without impacting anterograde SR through the SFA using the present protocol. However, the mixed SR pattern changes induced with the thigh cuff may be more reflective of the complex SR changes commonly observed with enhanced vascular tone.

The same group who previously presented an isolated retrograde stimulus in the brachial artery recently examined the effects of an altered SR profile on both brachial and SFA FMD but were not able to replicate their previous observations of an exclusive increase in retrograde SR [19]. In this recent study, cuff inflation to 60 mmHg resulted in mixed SR effects with increases in both anterograde and retrograde SR through both the SFA and the brachial artery [19]. In agreement with their previous work, they reported a correlation between change in FMD% and change in retrograde SR, and a decrease in FMD% in both arteries. They combined these findings with their initial study results and suggested that a threshold for external pressure exists between 30 and 50 mmHg; however it is difficult to conclude that this threshold is the same for the SFA as the initial study included the brachial artery only. In our current study we found a greater relative retrograde SR response to 100 mmHg vs. 75 mmHg evidenced by a decrease in mean SR at 100 mmHg. Future studies should explore a variety of cuff pressures and intervention lengths to ascertain whether creating an exclusive retrograde stimulus is possible through the SFA. Future studies should also calculate OSI when assessing altered SR patterns as it may be that simply evaluating the change in retrograde SR is not sufficient to assess the complex relationship between SR and endothelial function.

When calculating peripheral artery SR in humans it is assumed that the blood velocity profile is parabolic in shape; a recent study in humans by Ade et al. (2012) reported parabolic profiles for mean, anterograde and retrograde phases at rest, but during an increased downstream resistance intervention (cold pressor test), the mean and anterograde components exhibited a plug-like profile (MBV/PBV>0.5) [9]. In the present study we observed a sharpened retrograde parabolic profile (ratio <0.5), a plug-like mean profile (ratio>0.5), and a parabolic anterograde profile (ratio  = 0.5) at rest. During the cuff inflation intervention, we observed an increase in both anterograde and retrograde ratios; the retrograde profile remained a sharpened parabolic shape, while anterograde became a plug-like profile. The disparity between study results may be due to a number of factors: a) our intervention was local (on the thigh), whereas the previous study used a cold pressor test on the hand to induce sympathetic activation; b) our intervention assessed blood velocity profiles in the SFA, whereas the previous study assessed the common femoral artery, likely introducing variant responses due to the proximity of our measurements to the common femoral artery bifurcation; and c) our analysis included anterograde and retrograde blood velocity throughout the entire cardiac cycle, whereas the previous study only considered the first two phases of the cardiac cycle (Figure 1). Caution should be employed when interpreting the results from Ade et al. due primarily to the last rationale. Our work extends the knowledge in the field regarding true blood velocity shape across the entire cardiac cycle; however our findings of non-parabolic profiles suggests some error may be introduced into SR calculations. Future work should examine each phase of the cardiac cycle separately for blood velocity shape.

Limitations of our study include sample size and method of data acquisition. Although we saw significant results with the current sample size, it is possible the results would be more pronounced with a larger sample size. Our method of data acquisition was such that we were not able to simultaneously collect FMD measures on the cuffed and non-cuffed leg. Post-FMD measurement on the non-cuffed control leg was conducted after data collection on the cuffed leg (approximately 15-minutes following the cuff inflation intervention); theoretically FMD in this contra-lateral leg could have also been depressed immediately following the 30-minute intervention and then returned to baseline after 15-minutes. However, our SR data in the control leg were obtained during the cuff intervention and showed no changes in local flow environments as a result of the contralateral cuff inflation. In addition, it has been shown that alterations in FMD are persistent for up to 2-hours post-exercise [31]. Several other studies looking at endothelial health changes following various acute exercise interventions found alterations were still evident 30-minutes to 1-hour post-intervention [32], [33]; it is therefore unlikely that FMD in the control leg would have been attenuated and then returned to baseline within 15-minutes.

This study directly advances the current knowledge about the influence of modifications in oscillatory SR on arterial function and presents novel data about the regulation of endothelial function in clinically important lower limb arteries. Endothelial dysfunction is common in conditions where there is increased vascular tone, such as age, obesity, and hypertension. Chronic changes in endothelial function may be related to alterations in both SR magnitude and pattern; effective interventions to improve vascular health should take into account these regulatory mechanisms. Future studies should explore the acute effects of inducing an exclusively retrograde shear stimulus on endothelial function in the lower extremity in healthy as well as clinical populations.
